# Transcriptome analysis of thermogenic *Arum concinnatum* reveals the molecular components of floral scent production

**DOI:** 10.1038/srep08753

**Published:** 2015-03-04

**Authors:** Yoshihiko Onda, Keiichi Mochida, Takuhiro Yoshida, Tetsuya Sakurai, Roger S. Seymour, Yui Umekawa, Stergios Arg Pirintsos, Kazuo Shinozaki, Kikukatsu Ito

**Affiliations:** 1 Biomass Research Platform Team, Biomass Engineering Program Cooperation Division, RIKEN Center for Sustainable Resource Science, Kanagawa, Japan; 2 Kihara Institute for Biological Research, Yokohama City University, Kanagawa, Japan; 3 Gene Discovery Research Group, RIKEN Center for Sustainable Resource Science, Kanagawa, Japan; 4 Integrated Genome Informatics Research Unit, RIKEN Center for Sustainable Resource Science, Kanagawa, Japan; 5 School of Biological Sciences, University of Adelaide, Australia; 6 United Graduate School of Agricultural Science, Iwate University, Morioka, Japan; 7 Department of Biology and Botanical Garden, University of Crete, Heraklion, Greece; 8 Cryobiofrontier Research Center, Iwate University, Morioka, Japan

## Abstract

Several plant species can generate enough heat to increase their internal floral temperature above ambient temperature. Among thermogenic plants, *Arum concinnatum* shows the highest respiration activity during thermogenesis. However, an overall understanding of the genes related to plant thermogenesis has not yet been achieved. In this study, we performed *de novo* transcriptome analysis of flower organs in *A. concinnatum*. The *de novo* transcriptome assembly represented, in total, 158,490 non-redundant transcripts, and 53,315 of those showed significant homology with known genes. To explore genes associated with thermogenesis, we filtered 1266 transcripts that showed a significant correlation between expression pattern and the temperature trend of each sample. We confirmed five putative alternative oxidase transcripts were included in filtered transcripts as expected. An enrichment analysis of the Gene Ontology terms for the filtered transcripts suggested over-representation of genes involved in 1-deoxy-d-xylulose-5-phosphate synthase (DXS) activity. The expression profiles of DXS transcripts in the methyl-d-erythritol 4-phosphate (MEP) pathway were significantly correlated with thermogenic levels. Our results suggest that the MEP pathway is the main biosynthesis route for producing scent monoterpenes. To our knowledge, this is the first report describing the candidate pathway and the key enzyme for floral scent production in thermogenic plants.

Among the large number of poikilothermic plant species, several can raise their internal body temperature to a level higher than the ambient temperature. In fact, descriptions of thermogenic plants have a long history extending back to the 18th century; for example, thermogenesis of the inflorescence in the European arum lily was described by de Lamarck (1778; cited in Ref. 1) and the true flower of *Nelumbo nucifera* was described by Miyake^2^ to warm appreciably; however, this extraordinary phenomenon in plants was not studied extensively for a long time. Ever since the 1970s, *Philodendron selloum*^3^, *Symplocarpus foetidus*^4^, *Arum maculatum* and *A. italicum*^5^, *Symplocarpus renifolius*^6^, *Nelumbo nucifera*^7^, *Dracunculus vulgaris*^8^, *Helicodiceros muscivorus*^9^, *Amorphophallus titanum*^10^, and *Arum concinnatum*^11^ were analyzed and reported as thermogenic plants. The thermogenic plants are divided into two types based on the pattern of heat production, i.e., thermoregulatory or not. Thermoregulatory plant species can regulate their internal body temperature during a few days of thermogenesis even when ambient temperature fluctuates, however, non-thermoregulatory plant species cannot regulate and thermogenesis occurs transiently for only a few hours. Thus, it is apparent that the rate of heat production must be opposite to flower temperature change in thermoregulatory plants (e.g. the rate of heat production must increase as flower temperature decreases)^12^. Only *S. foetidus*^4^,^13^,^14^, *S. renifolius*^15^, *D. vulgaris*^8^, *P. selloum*^3^,^16^, and *N. nucifera*^7^ are definitely thermoregulatory species. There are likely to be others, particularly among the many *Philodendron* species.

There are three major benefits of heat production in thermogenic plants: (1) to protect the reproductive process from low temperatures^4^,^17^, (2) to reward insect pollinators in the flower by providing a warm environment^18^, and (3) to attract insect pollinators by volatilizing floral scent compounds^19^. All these roles are important for understanding the biological significance of thermogenesis in plants, which involves a substantial energy cost. However, little is known about the molecular mechanisms underlying these phenomena.

Previous studies in thermogenic plants were focused on enzymatic regulation to generate heat in plants. A well-known key player of thermogenesis in plants is mitochondrial alternative oxidase (AOX), which is also found among fungi and nematodes^20^,^21^,^22^,^23^,^24^. AOX accepts electrons from the ubiquinone pool and uses them to reduce oxygen to water^25^. In contrast to cytochrome *c* mitochondrial terminal oxidase, AOX bypasses complexes III and IV and conserves energy by not pumping protons. Thus, the free energy generated by the flow of electrons from the ubiquinone pool to AOX is generally believed not to lead to ATP synthesis, but to be released as heat^26^. In addition to AOX, plants have unique energy-dissipating systems: rotenone insensitive type II NAD(P)H dehydrogenases (NDA and NDB) in both the inner and outer surfaces of the mitochondrial inner membrane^27^. NDA and NDB are classified as type II, whereas rotenone sensitive and proton pumping complex I are classified as type I. NDA and NDB oxidize NAD(P)H and transport the electrons to ubiquinone without pumping protons, bypassing complex I^27^; however, the involvement of these molecules in thermogenesis has only been recently investigated in *A. maculatum*^28^. In mammals, the mitochondrial uncoupling protein (UCP) has been shown to play a crucial role in thermogenesis^29^. Although mammalian mitochondria contain neither AOX nor type II NAD(P)H dehydrogenases, UCP resides in the inner mitochondrial membrane. The presence of UCPs in plants gained widespread acceptance with the identification of the first plant UCP-encoding gene from potato^30^; since then, UCP-encoding genes have been identified from a wide range of plant species^31^,^32^. Overall, previous reports indicate that key players of plant thermogenesis differ depending on the plant species and include not only AOX but also NDA, NDB, and UCP^28^,^33^,^34^,^35^,^36^,^37^. Detailed molecular mechanisms (e.g., expression of protein and regulation of activity) of AOX and UCP have been reported^28^,^33^,^36^,^37^,^38^,^39^,^40^; however, an overall understanding of the genes related to plant thermogenesis has not yet been achieved.

Among thermogenic species, *A. concinnatum* is non-thermoregulatory, but shows the highest mass-specific respiration rates during heat production^11^,^41^. *A. concinnatum* produces an inflorescence, which consists of a spadix with male florets and female florets in the floral chamber surrounded by the spathe. Above the floral chamber a large appendix becomes exposed when the spathe opens in the afternoon and evening of the pistillate stage of blooming (called D-day). This is accompanied by a single bout of intense warming of the appendix to over 30°C. When the appendix temperature reaches its peak, accompanied by spathe opening and scent volatilization, several insect families of Diptera (e.g., Sphaeroceridae, Sciaridae, and Drosophilidae) and a family of Coleoptera (Ceratopogonidae) are attracted to the inflorescence^42^. The insects remain in the floral chamber where the thermogenic male florets provide warmth overnight. About 24 hours after the pistillate stage, the staminate stage results in pollen production after which the male florets cease thermogenesis. Although male florets are more thermogenic on a mass-specific basis than the appendix, they weigh much less (0.4 g vs. 14.5 g), and therefore do not reach a temperature as high as the appendix (e.g., 34°C for the appendix, 25°C for male florets, and 22°C for female florets under an ambient temperature condition of 22°C)^11^. The mean rate of heat production of the whole appendix is 1076 mW, the male florets 157 mW and female florets negligible heat. These differences in heat production levels among the floral parts are convenient for comparing the molecules related to heat production in an individual.

In this study, to develop a global view of the transcriptome underlying thermogenesis and to elucidate the genes involved in heat production in plants, we performed *de novo* transcriptome analysis of the flower organs in *A. concinnatum*. Because the genome size of this plant was reported as 23,445 Mbp^43^, it is not practical to perform whole-genome sequencing. Thus, RNA-seq with a reference sequence could not be applied. *De novo* transcriptome analysis is becoming a useful tool to gain sequence information regarding expressed genes and expression patterns without a genome sequence as a reference^44^,^45^,^46^. Based on the RNA-seq dataset of *A. concinnatum*, we present comparative properties of the transcriptome among stages (on pre-D-day and on D-day) and tissues (male florets for intermediate heating level, female florets for never heating, and the appendix for most thermogenic) with different thermogenic properties. We also predict putative genes that are associated with thermogenic levels of the flower organs based on a co-expression analysis. Herein, we present the first comprehensive view of the transcriptome to provide new insights into plant thermogenesis, especially with respect to a metabolic process of volatilizing floral scent compounds.

## Results

### *De novo* transcriptome assembly of flower organs in *A. concinnatum*

**Figure 1:**
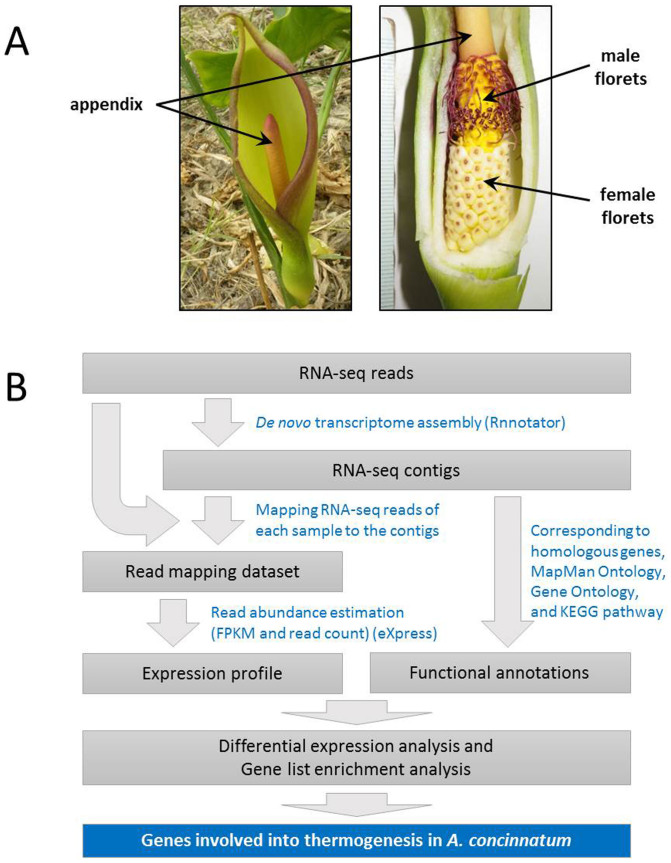
Schematic overview of the transcriptome analysis of thermogenic inflorescences of *A. concinnatum*. (A) Anatomical representation of the thermogenic flower organs of *A. concinnatum*. (B) Analytical workflow of a *de novo* RNA-seq analysis used in this study.

Transcriptome analysis was performed using RNAs extracted from three organs of the inflorescence (male florets, female florets, and the appendix) at two stages (pre-D-day and D-day) (Figure 1A). These organs and stages exhibit different temperatures^11^. In this sampling, we confirmed the heating level of the appendix in comparison with air temperature; the appendix temperature was elevated to more than 31°C at D-day. By using an Illumina Hiseq2000 platform, 26 to 32 million paired-end reads were obtained in each sample, and these passed a filtering process as cleaned reads (Supplementary Table S1). In total, 180 million paired-end reads representing 34.2 Gbase cleaned nucleotides were used to the assembly process of sequence by using the Rnnotator program^47^ to generate a non-redundant dataset of contig sequences. The forward reads were mapped to the dataset of contig sequences as the reference transcripts using a bowtie program^48^ to estimate the abundance of reads corresponding to each transcript in each sample. In the mapping, 44% to 59% of reads were mapped to the reference transcripts (Supplementary Table S1). Based on the mapping results, the fragments per kilobase of transcript per million mapped reads (FPKM) value of each gene in each sample was computed as a dataset of global expression profiles by using the eXpress program (Figure 1B).

### Overview of the transcriptome in thermogenic inflorescences

**Figure 2:**
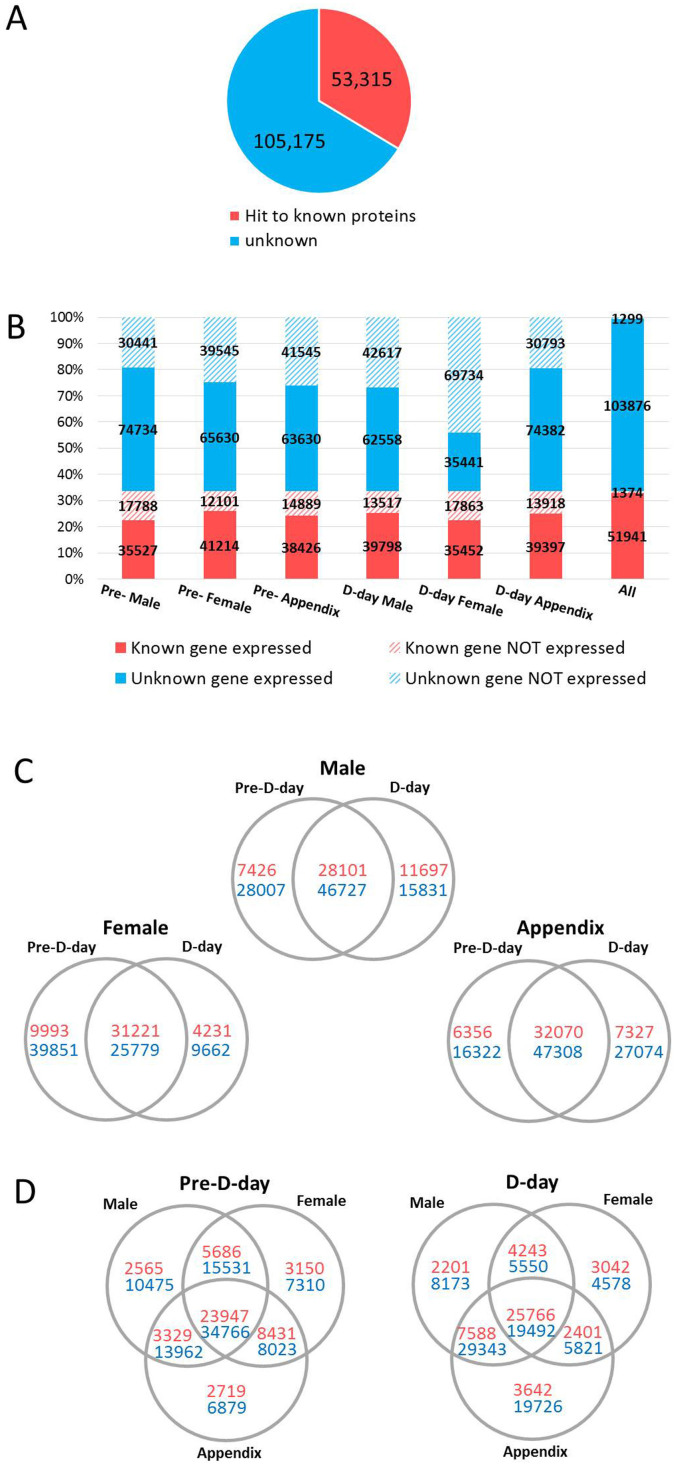
Overview of expressed transcripts in the thermogenic inflorescences. (A) Proportion of known transcripts that showed sequence similarity with NCBI nr entries or proteins of *Arabidopsis* (TAIR10). (B) Number of expressed transcripts (FPKM ≥ 1) in each tissue and at least one tissue RNA sequenced in this study. (C) Organ-wise comparison of specificity and commonality of expressed transcripts. (D) Specificity and commonality of expressed transcripts among organs in pre-thermogenic and thermogenic stages.

The *de novo* transcriptome assembly represents, in total, 158,490 non-redundant transcripts identified in the *A. concinnatum* inflorescence (Figure 2A). To classify the transcripts to putative homologs of known genes or others, we performed a sequence similarity search against known protein sequence datasets (NCBI nr and *Arabidopsis* proteome in TAIR10) by using the BLASTx program (E < 10^−5^). The BLASTx search showed 53,315 (33.6%) transcripts with significant sequence similarity with entries of NCBI nr and/or those of TAIR10 (known), and the others 105,175 (66.4%) transcripts did not provide any significant hits (unknown) (Figure 2A). The sequence length distribution differed between the known and unknown transcripts. The sequence length of the known transcripts ranged to more than 4 kbp. On the other hand, the unknown transcripts were less than 2.2 kbp long, and most transcripts were less than 600 bp long (Supplementary Figure S1). Throughout this study, we defined transcripts with FPKM values ≥ 1 in at least one sample as the threshold of an expressed gene. Figure 2B shows numbers and proportions of transcripts of known genes and those of unknown genes that are expressed or not expressed in each sample. In every sample, more than 35,000 transcripts of putative homologs of known genes were identified with FPKM ≥ 1. Most sequences without any significant hits to known proteins also showed significant expression, with FPKM ≥ 1 in each sample (Figure 2B). Organ-wise comparisons of expressed transcripts between pre-D-day and D-day in male florets, female florets, and the appendix showed commonly and specifically expressed transcripts in each organ at stages with different thermal states (Figure 2C). For instance, in thermogenic male florets and the appendix at D-day, 11,697 and 7327 transcripts of known genes were specifically expressed, respectively. The distribution of expressed transcripts among the three organs showed specific expression of transcripts for each organ at pre-D-day and D-day (Figure 2D). More than 8400 transcripts of known genes were commonly expressed in the female florets and the appendix only at pre-D-day. On the other hand, 7500 transcripts of known genes were commonly expressed in the male florets and the appendix only at the D-day. These results of the *de novo* transcriptome assembly represent a number of genes that are specifically co-expressed in each of the thermogenic organs (male florets and appendix) of the inflorescence at D-day in *A. concinnatum* (Figure 2D). In addition to the sequence similarity searches, we performed further functional annotation of the *A. concinnatum* transcripts to collect clues as much as possible to infer gene function (Supplementary Figure S2).

### Differentially expressed genes at pre-D-Day and D-day

**Figure 3:**
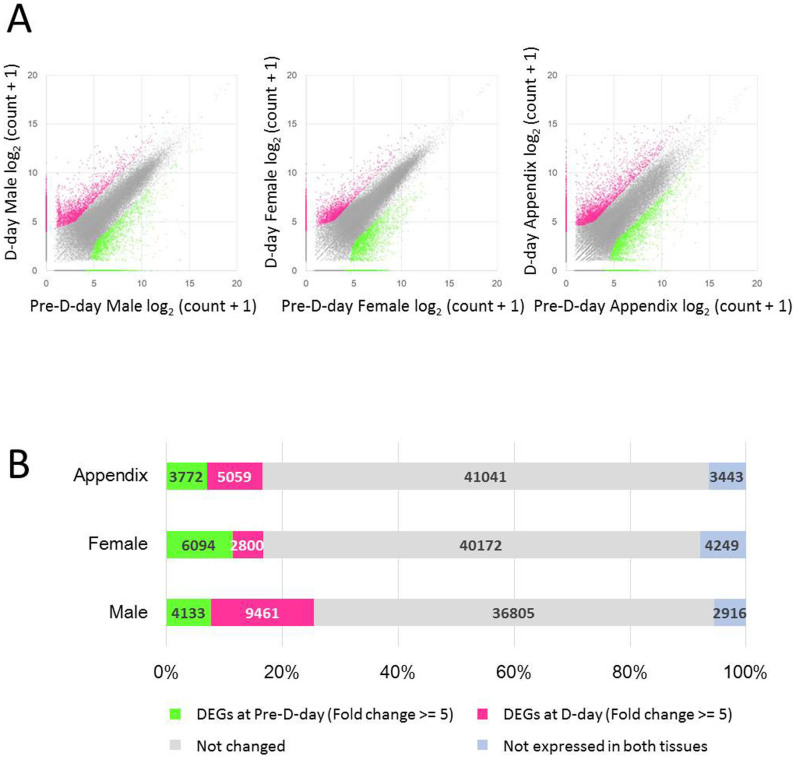
Differentially expressed genes (DEG) in the flower organs. In total, 53,315 transcripts that showed significant homology with known protein sequences were analyzed. (A) Scatter plots of pairwise combinations of tissues with selected DEGs (colored) in the male and female florets and the appendix. DEGs were selected using DEGseq with Fishers exact test (*P* < 10^−5^, fold change ≥ 5) (B) Numbers of DEGs in each combinations of organs.

A normalized RNA-seq read count dataset from the eXpress program^49^ of transcripts of known genes was used to identify genes that are differentially expressed in each organ between pre-D-day and D-day. Differentially expressed genes (DEGs) were determined using a false discovery rate (FDR) threshold corrected *P* value < 10^−5^ using Fisher's exact test and a fold change ≥ 5 with the normalized read count mapped to each transcript. These were computed using the DEGseq program (Figure 3A). Based on the DEG analysis, 5059 transcripts were up-regulated in the appendix at D-day, whereas 3772 transcripts were up-regulated in the appendix at pre-D-day. In the female florets, 2800 and 6094 transcripts were up-regulated at D-day and pre-D-day, respectively (Figure 3B). In the male florets, 9461 and 4123 transcripts were up-regulated at D-day and pre-D-day, respectively.

**Figure 4:**
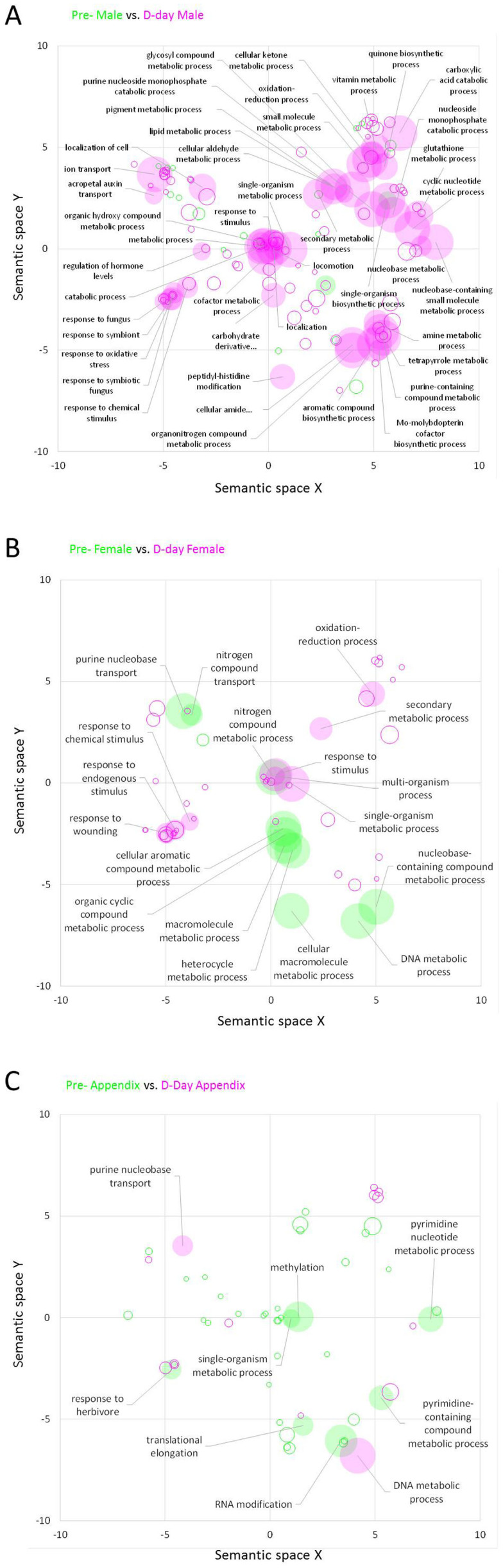
Significantly enriched gene functions in DEGs found in combinations of pre-D-day and D-day tissues. Enriched gene functions were projected to a 2D semantic space of the gene ontology, biological process. Green circles and pink circles correspond to significantly enriched GO terms found in DEG genes at pre-D-day and D-day, respectively. The circle size represents the −log_10_ transformed FDR in REVIGO analysis. Circles depicted by filled color show significantly enriched GO terms with FDR < 10^−5^. (A) Male florets at pre-D-day and D-day, (B) Female florets at pre-D-day and D-day, and (C) Appendix at pre-D-day and D-day. The semantic space was generated by the REVIGO web service with all enriched GO terms found in the DEG gene sets.

Gene Ontology (GO) enrichment analysis was performed to infer particular functional categories of genes over-represented in up-regulated DEGs in each sample. Various GO terms were significantly enriched in each group of DEGs with a false discovery rate (FDR) threshold corrected *P* value < 0.05 using Fisher's exact test on the BLAST2GO program (Supplementary Table S2). In particular, the number of GO terms that were enriched are 120 in pre-D-day male, 52 in pre-D-day female, 189 in pre-D-day appendix, 890 in D-day male, 184 in D-day female, and 92 in D-day appendix tissues (Supplementary Table S2). These GO terms were enriched from DEGs as shown in Figure 3B. To summarize the significantly enriched GO terms along with semantic similarities, the enriched GO terms of the biological process ontology were represented on the 2D semantic space (Figure 4). In the male florets, 19 GO terms at pre-D-day and 120 GO terms at D-day were significantly enriched. In the male florets at D-day, genes involved in a number of biological processes were particularly up-regulated. The summarization on the 2D semantic space presents semantic clusters of GO terms related to the response to some biological stimulus, and the metabolic processing of various primary and secondary metabolites in the male florets at D-day (Figure 4A). In the female florets, 11 GO terms at pre-D-day and 42 GO terms at D-day were significantly enriched. In the female organs, GO terms of some metabolic processes were particularly over-represented at pre-D-day, whereas GO terms related to response to stimulus were weakly over-represented at D-day (Figure 4B). In the appendix, 39 GO terms at pre-D-day and 14 GO terms at D-day were significantly enriched. Although not many GO terms were particularly over-represented in the appendix, GO terms of pyrimidine nucleotides or compound metabolic processes were enriched at pre-D-day, whereas those of purine nucleobases or compound metabolic processes were enriched at D-day (Figure 4C). The comparative GO enrichment analyses between pre-D-day and D-day in each organ represent differential up-regulation of genes involved in various processes behind the flowering of *A. concinnatum*.

### Co-expressed genes with their associated temperature profiles

**Figure 5:**
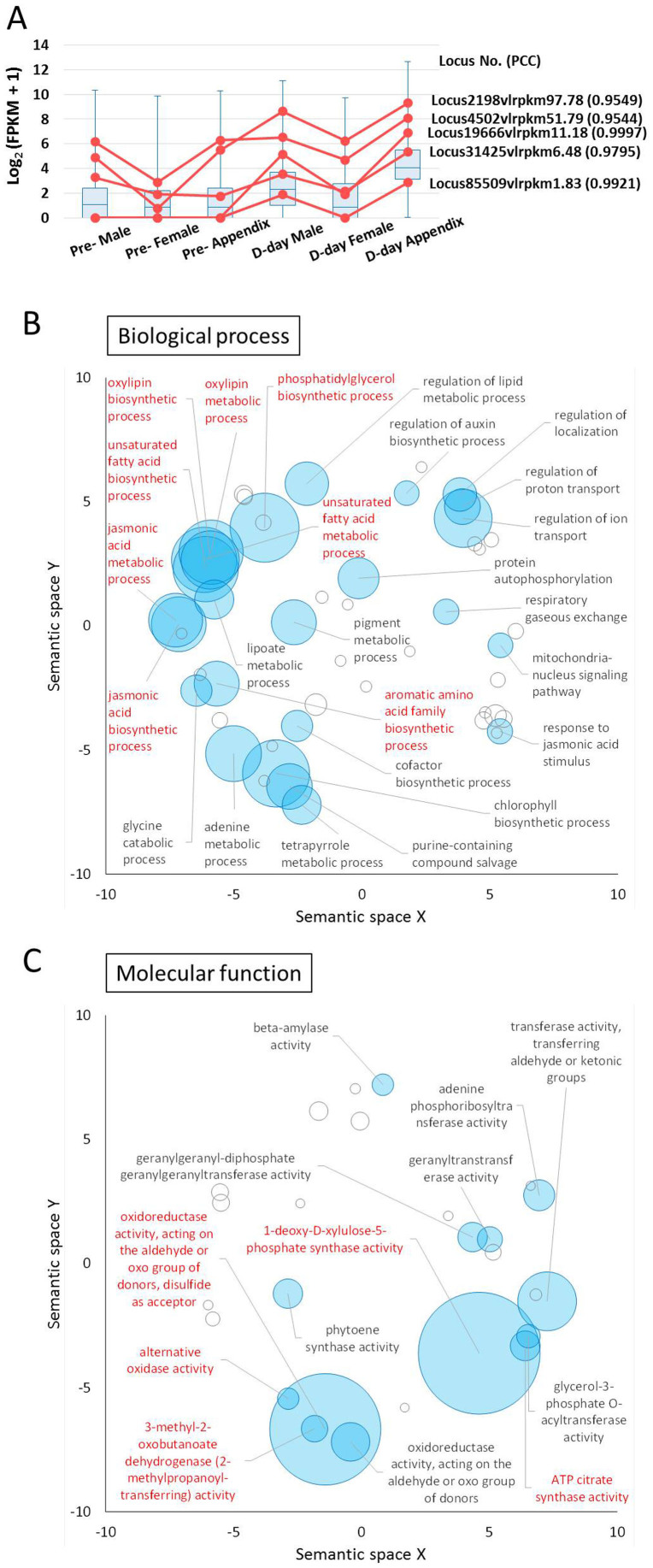
Genes with expression patterns that correlated with the temperature pattern. (A) Expression trend of 1266 transcripts whose expression patterns were highly correlated with temperature patterns in each organ (Pearson's correlation coefficient (PCC) ≥ 0.95). Red colored line plots are expression patterns of homologs of AOX encoding genes. (B) and (C) Enriched gene functions of GO categories of the correlated genes with the temperature pattern projected onto a 2D semantic space. The circle size represents the −log_10_ transformed FDR in REVIGO analysis. Circles depicted by filled color show significantly enriched GO terms with FDR < 10^−5^. (B); GO categories related to biological process. (C); GO categories related to molecular function.

To explore genes associated with *A. concinnatum* thermogenesis, we filtered transcripts from 53,315 known transcripts that showed a significant correlation between expression pattern and the temperature trend of each sample. As a result, 1266 known transcripts (2.37%) that showed a strong correlation (Pearson's correlation coefficient (PCC) ≥ 0.95) with the temperature trend were labeled with an ID that begins with “Locus” (Supplementary Table S3). A box plot summarizing the expression of these transcripts agreed with the temperature trend, which are slightly heated in male florets and well heated in the appendix at D-day (Figure 5A). On the other hand, among 105,175 unknown transcripts, 3195 transcripts (3.72%) showed a significant correlation between expression pattern and the temperature trend of each organ. There is a possibility that a number of unknown transcripts from related gene families are associated with the heat production.

#### AOX-encoding genes

The 1266 transcripts that correlated with temperature include those of five genes (Locus19666v1rpkm11.18, Locus2198v1rpkm97.78, Locus31425v1rpkm6.48, Locus4502v1rpkm51.79, and Locus85509v1rpkm1.83) putatively encoding AOX. Transcripts of Locus19666 and Locus85509 were especially correlated with the heat pattern, being not expressed in any organ at pre-D-day, and mildly expressed in male florets, and having highest expression in the appendix at D-day. Transcripts of three other genes (Locus2198, Locus31425, and Locus4502) were expressed in the organs even at pre-D-day (Figure 5A). In this correlation analysis, only AOX showed significantly higher correlation; other genes for mitochondrial energy-dissipating enzymes (namely UCP, NDA, and NDB) were not significantly correlated with the thermogenic levels of each organ (Table 1). These results suggest that gene expression for AOX plays a critical role in thermogenesis of *A. concinnatum*, and UCP, NDA and NDB may act in a rather ubiquitous fashion in this non-thermoregulatory plant. In a previous study, two mRNAs encoding AOX in *A. concinnatum* were reported (AB485993 for *AcoAOX1a* and AB485994 for *AcoAOX1b* in DDBJ)^39^. In *A. maculatum*, another *Arum* species distributed around Europe, at least seven transcripts for *AOX* were cloned^50^. Our transcriptome analysis revealed at least seven independent transcripts putatively encoding AOX. This result suggests that there are unidentified *AOX* homologs other than *AcoAOX1a* and *1b* in *A. concinnatum*.

#### Enriched GO terms

GO enrichment analysis was performed to infer particular functional categories of genes over-represented in the 1266 selected transcripts. In total, 131 GO terms were significantly enriched in this group of transcripts (Supplementary Table S4). The summarization on the biological process ontology of the 2D semantic space presents semantic clusters related to metabolic processes of the unsaturated fatty acid, oxylipin, and jasmonic acid, particularly (Figure 5B). On the molecular function ontology, we observed particularly enriched categories of oxidoreductase and alternative oxidase activity because of putative AOX-encoding genes. In addition, we observed 1-deoxy-d-xylulose-5-phoshate synthase (DXS) activity was especially enriched, with the lowest corrected *P* value (5.85 × 10^−19^) in the tested group (Figure 5C).

#### DXS-encoding genes

**Figure 6:**
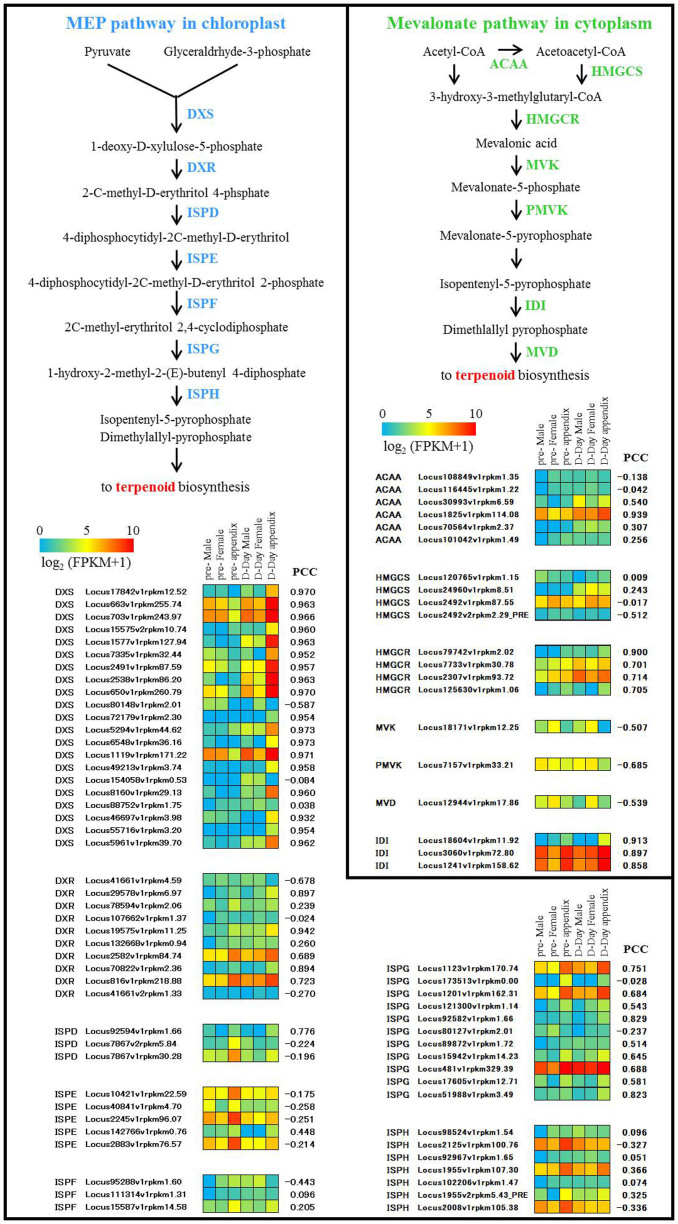
Expression profiles of genes involved in terpenoid biosynthesis. Red and blue colors in the tiled panel represent higher and lower gene expression levels, respectively. Each of Pearson's correlation coefficient (PCC) with the inflorescence temperature pattern was also shown at the right of the tiled panel.

In the transcripts with known protein hits, we identified 21 putative homologs of the *Arabidopsis thaliana* DXS gene (At4g15550). In the 1266 selected transcripts, 24 entries were classified into the GO category of DXS activity, which includes 17 putative DXS encoding transcripts. This means expression of most of the putative *DXS* genes was correlated with the thermogenic level (heating pattern) of the flower organs. DXS is the first enzyme of the methyl-d-erythritol 4-phosphate (MEP) pathway, and plays a major role in the regulation of this pathway^51^. It is well known that there are two metabolic pathways for terpenoids biosynthesis: the MEP pathway (non-mevalonate pathway) and the mevalonate pathway. Therefore, we examined the expression profiles of transcripts putatively encoding enzymes involved in both these pathways based on the RNA-seq dataset (Figure 6). The expression pattern of each of 17 transcripts encoding putative DXS showed strong correlation with the temperature pattern of the flower organs and stages, and showed extremely elevated expression in the appendix at D-day compared with pre-D-day. For example, the expression Locus2538 was more than a thousand-fold increased from pre-D-day to D-day in the appendix, with strong correlation (Pearson's correlation coefficient (PCC) = 0.963). On the other hand, only one transcript of the mevalonate pathway (Locus 1825, encoding the first step enzyme ACAA) showed strong correlation between its expression and temperature (PCC = 0.939). Previous studies reported that DXS catalyze one of the late-limiting reaction of the MEP pathway for isoprenoid biosynthesis in *Arabidopsis thaliana*^52^ and *Solanum lycopersicum*^53^,^54^. Our *de novo* transcriptome analysis revealed that the expression patterns and particular up-regulation of DXS were strongly correlated with thermogenesis. These results also suggest that this step might be a rate-limiting step in biosynthesis of terpenoids leading to volatile compounds for the scent from this plant.

## Discussion

### *De novo* assembly of the *A. concinnatum* transcriptome

*De novo* transcriptome analysis for thermogenic plants provides an opportunity to discover genes involved in floral thermogenesis and scent production. It has become a useful approach to rapidly build comprehensive sequence resources of expressed genes without whole genome sequencing and to examine genome-scale gene expression patterns of RNA samples of interest. Thus, *de novo* transcriptome analysis has been applied to a wide range of plant species such as medicinal plants, crops, and plants with particular biological systems^55^,^56^,^57^. For organisms with large, complex genomes containing repetitive sequences and polyploidy, including thermogenic plants, RNA-seq based *de novo* transcriptome analysis is a promising procedure to examine properties of a transcriptome as a proxy for the whole genome^44^.

*De novo* transcriptome assembly contained more than 150,000 transcripts, even though only non-redundant transcripts were included in this study. Functional prediction of these transcripts resulted in only 33.6% of the transcripts being associated with at least one homologous protein; no proteins were identified for the remaining transcripts. Possibly, the dataset of assembled transcripts includes many noisy sequences, or some non-coding transcripts such as functional non-coding RNAs and expressed transposable elements. With mapping of RNA-seq reads and estimation of gene expression, most of the unknown transcripts also showed a significant level of gene expression, with FPKM values ≥ 1, as well as expression in several tissues (Figure 2). The large-scale RNA-seq reads should become a significant sequence resource that should facilitate identifying homologous counterparts in *A. concinnatum* and could shed light on the largely unexplored transcriptome in thermogenic plants.

### Differential gene expression analysis

Differential gene expression analysis in this study identified a number of transcripts that were differentially expressed between pre-D-day and D-day. In total, 139 (19 at pre-D-day and 120 at D-day), 53 (11 at pre-D-day and 42 at D-day), and 53 (39 at pre-D-day and 14 at D-day) summarized GO terms in biological process were significantly enriched in the male florets, female florets, and the appendix, respectively (Figure 4). Interestingly, although more GO terms were enriched at D-day in the thermogenic male and non-thermogenic female florets compared with that at pre-D-day, fewer GO terms were enriched in the thermogenic appendix at D-day. This result suggests that a number of transcripts categorized into the same GO terms have been sufficiently prepared before thermogenesis, or less significance of over-representation of functional category of GOs due to a wider variety of transcripts in the appendix which organ produces remarkable heat in this plant.

Although we could not identify prominent DGEs in the appendix by a simple comparison between pre-D-day and D-day samples, we did identify genes that were significantly correlated with thermogenic levels among samples (Figure 5). First, we focused on AOX, a well-known energy-dissipating mitochondrial enzyme in thermogenic plants, as a positive control. Five putative *AOX* genes were significantly correlated with the temperature patterns of each sample. This expression pattern was consistent with that of a previous study in *A. concinnatum*^39^ and other thermogenic species such as *D. vulgaris*^34^ and *S. renifolius*^38^.

The GO categories that were most enriched related to the biological process involved in metabolism of the unsaturated fatty acids, oxylipin, and jasmonic acid (Figure 5B). Jasmonic acid, an oxylipin, is derived from the unsaturated fatty acid, linolenic acid, in the chloroplast membrane^58^,^59^,^60^. The jasmonic acid signaling pathway is involved in defense responses to injury and in programmed cell death and senescence^61^. When the appendix temperature rises and reaches its peak, accompanied by floral scent volatilization, several species of pollinator insects are attracted to the inflorescence^42^. In the only other study of the transcriptome in thermogenic plants, Ito-Inaba et al. demonstrated that genes involved in stress responses and protein degradation are up-regulated during the post-thermogenic stages in skunk cabbage (*S. renifolius*)^62^. Accordingly, the enriched gene expression for jasmonic acid metabolism seems to be related to programmed cell death and senescence, followed by energy-consuming thermogenesis, rather than to injury caused by attracted pollinator insects.

For molecular function, the most outstanding enriched GO category was 1-deoxy-d-xylulose-5-phosphate synthase activity (Figure 5C). The expression of *DXS* genes in heated organs, especially in the appendix, might be involved in the synthesis of terpenoids, depending on the temperature increases in the flower organs (Figure 6). As a distinctive phenomenon associated with thermogenesis in *A. concinnatum*, the flowers volatilize a strong dung/urine-like smell at D-day^42^. Metabolome analysis by gas chromatography/mass spectrometry (GC-MS) on the volatile compounds showed that terpenoids (3,7-dimethyl-1,6-octadine, 2,7-dimethyl-1,7-octadiene, and 3-7-dimethyl-1-octane) mainly account for the smell of *A.* *concinnatum*^42^,^63^. Because the transcripts of the first step of the mevalonate pathway showed lower correlation with the thermogenic level compared with transcripts of the first step of the MEP pathway (Fig. 6), the MEP pathway seems to be the main biosynthetic route for producing these three basic scent monoterpenes in this plant. To the best of our knowledge, this is the first report describing the candidate pathway and the key enzyme for floral scents in thermogenic plants.

### Evolution of thermogenic plants

Heat is a byproduct of metabolism in all flowering plants. In most plants, metabolic reactions are too slow to elevate tissue temperature^64^, or heat escapes very quickly because the tissue is too small to retain heat^41^. Irrespective of whether plants produce heat, the reproductive process is one of the most pivotal life events for all seed plants. In thermogenic plants, diverse relationships exist between the types of odor and the insect pollinators they attract. For example, *P. selloum* produces 4-methoxystyrene and 3,4-dimethoxystyrene and attracts the dynastid scarab beetle (*Cyclocephala variolosa*)^65^. *H. muscivorus* (dead-horse arum) produces oligosulphides, dimethyl mono-, di- and trisulphide and attracts flies^66^. *A. maculatum* produces indole and 2-heptanone^67^ and attracts drain flies (*Psychoda phalaenoides*)^68^. These observations suggest that heat production and the accompanying floral scent volatilization may have coevolved with the olfactory receptor of insect pollinators. As described above, thermogenic plants produce different floral scents and use different combinations of energy-dissipating systems for heat production to attract insect pollinators. Thermogenic plants have undergone a variety of evolutionary changes and has diversified with regard to reproduction. In conclusion, the present study firstly establishes a comprehensive and detailed relationship between global gene expression and reproductive processes, in particular, floral thermogenesis and scent production in *A. concinnatum*. With *de novo* transcriptome analysis, it is possible to shed light on the unknown molecular components of the metabolic pathway involved in plant thermogenesis and pollinating insect attraction.

## Methods

### Plant materials and temperature measurements

*A. concinnatum* plants were collected in May 2013 from a field situated near the village of Panormos, Crete Island, Greece. The appendix temperature was measured by thermistors placed into the tissue. Air temperature was measured in the same way. Three individuals were randomly selected and collected for following RNA extraction. Mean temperature of both appendix and air were 23.0 ± 0.0°C at pre-D-day, and mean temperature of appendix and air were 31.3 ± 0.5°C and 23.0 ± 0.0°C at D-day, respectively.

### RNA-seq analysis

Total RNA was extracted using NucleoSpin RNA Plant (MACHEREY-NAGEL) from flash-frozen male florets, female florets, and the appendix of *A. concinnatum* plants at pre-D-day and D-day from the three independent individuals. Quality and quantity of all total RNA samples were checked by using NanoDrop (Thermo Fisher Scientific) and a 2100 Bioanalyzer (Agilent). Before library construction, total RNAs from the three individuals were combined and analyzed as a sample. First-Strand cDNA synthesis was performed using SMARTer® Ultra™ Low Input RNA for Illumina® Sequencing – HV (Clontech), according to the manufacturer's instructions. Libraries for RNA-seq analysis were constructed using a Low Input Library Prep Kit (Clontech) with Indexing Reagents to be barcoded according to the manufacturer's instructions. Prepared libraries were sequenced with Hiseq2000 by using TruSeq SBS Kit v3-HS (Illumina). The dataset of the Illumina reads has been submitted to DDBJ Sequence Read Archive under the accession number DRA002584.

### *De novo* transcriptome assembly

The FASTX tool kit (http://hannonlab.cshl.edu/fastx_toolkit) was used to assess quality score distributions of the Illumina reads. We estimated the most appropriate k-mer size for the following *de novo* assembly by using the KmerGenie program (http://kmergenie.bx.psu.edu/). The Rnnotator program^47^ was used for the *de novo* assembly of the Illumina RNA-seq reads with the estimated K-mer = 31. The significantly expressed transcripts were filtered with a threshold FPKM values ≥ 1, as described below. The assembled transcripts with length ≥ 200 bp were used as the sequence dataset of reference transcripts.

### Functional annotations

To predict the function of the assembled transcripts, we performed a similarity search of the assembled sequences as queries by using the NCBI BLASTx program^69^, with a threshold E value less than 10^−5^ against protein databases of the NCBI nr and the *A. thaliana* proteome dataset of TAIR10^70^. Functional classification of the assembled transcripts with the MapMan ontology was performed using the web-based Mercator pipeline^71^ (http://mapman.gabipd.org/web/guest/app/Mercator). Functional classification of the assembled transcripts with Gene Ontology was performed by integrated use of the BLAST2GO program with BLASTx search (against the Viridiplantae subset of NCBI nr, with a threshold E value less than 10^−6^) and InterProScan (default settings on the BLAST2GO) and TAIR GO assignment based on BLASTx search to the proteome dataset of TAIR10 described above. Functional classification of the assembled transcripts with KEGG enzymes was performed using the KAAS web service (http://www.genome.jp/tools/kaas/) with bidirectional mode and using the KEGG assignment function provided by BLAST2GO.

### Differential gene expression analysis

The Illumina reads were mapped to the assembled transcript sequence set using the bowtie program (ver. 1.0.0)^48^ with a default setting in the single end mode. Expression of each transcript was quantified using eXpress^49^. We defined transcripts with FPKM ≥ 1 in at least one tissue as significantly expressed. Based on a normalized read count mapped to each transcript, the DEGseq, an R package^72^ was used to test and identify differentially expressed transcripts with a FDR-corrected *P* value from Fisher's exact test < 10^−5^ and fold change ≥ 5.

### GO Enrichment analysis

GO enrichment analyses of selected transcripts were performed using a GO enrichment analysis function provided by BLAST2GO with a threshold FDR-corrected *P* value from Fisher's exact test < 0.05. Summarization of GO terms based on their semantic similarities and representation of summarized GOs on the 2D semantic space was performed on the REVIGO web service (http://revigo.irb.hr/)^73^.

## Supplementary Material

Supplementary Information Supplementary Table S1

Supplementary Information Supplementary Table S2

Supplementary Information Supplementary Table S3

Supplementary Information Supplementary Table S4

Supplementary Information Supplementary Figures S1 and S2

## Figures and Tables

**Table 1:** The results of expression and correlation analysis of energy dissipating system found in this study.

ID	Expression (FPKM)						
alternative oxidase (AOX)	Pre-D-day Male	Pre-D-day Female	Pre-D-day Appendix	D-day Male	D-day Female	D-day Appendix	PCC
Locus104409v1rpkm1.43	2.92E-01	1.73E+00	2.01E+00	2.06E+00	0.00E+00	6.94E-01	−0.086
Locus113826v1rpkm1.27	1.08E+01	7.67E+00	1.32E+01	0.00E+00	0.00E+00	4.61E+00	−0.294
Locus19666v1rpkm11.18	0.00E+00	0.00E+00	0.00E+00	3.44E+01	2.66E+00	1.16E+02	1.000
Locus2198v1rpkm97.78	2.85E+01	7.10E-01	4.42E+01	3.97E+02	7.46E+01	6.35E+02	0.955
Locus2221v1rpkm96.81	5.54E+01	2.34E+01	8.18E+01	1.06E+02	3.13E+01	9.09E+01	0.573
Locus31425v1rpkm6.48	8.65E+00	2.72E+00	2.33E+00	1.06E+01	3.36E+00	3.91E+01	0.980
Locus3229v1rpkm69.53	6.88E+01	2.75E+01	1.21E+02	8.68E+01	6.81E+01	1.29E+02	0.617
Locus4502v1rpkm51.79	7.08E+01	6.33E+00	7.73E+01	9.12E+01	2.45E+01	2.72E+02	0.954
Locus52656v1rpkm3.43	5.29E+00	3.51E+00	0.00E+00	9.58E+00	0.00E+00	8.26E+00	0.658
Locus85509v1rpkm1.83	0.00E+00	0.00E+00	0.00E+00	2.68E+00	0.00E+00	6.28E+00	0.992
Locus87119v1rpkm1.79	0.00E+00	0.00E+00	0.00E+00	0.00E+00	5.75E+00	6.76E+00	0.621

uncoupling protein (UCP)	Pre-D-day Male	Pre-D-day Female	Pre-D-day Appendix	D-day Male	D-day Female	D-day Appendix	PCC
Locus1928v1rpkm109.42	1.70E+01	2.96E+00	2.09E+01	1.82E+02	1.27E+01	7.97E+01	0.476
Locus56578v1rpkm3.14	2.19E+01	1.02E+01	1.06E+01	3.20E+00	2.64E+01	3.86E+00	−0.611
Locus8198v1rpkm28.98	5.31E+01	5.29E+01	1.13E+01	2.80E+01	3.86E+01	9.98E+00	−0.610

rotenone insensitive type II NAD(P)H dehydrogenases (NDA and NDB)	Pre-D-day Male	Pre-D-day Female	Pre-D-day Appendix	D-day Male	D-day Female	D-day Appendix	PCC
Locus110607v1rpkm1.32	0.00E+00	8.52E-01	3.43E+00	4.05E+00	4.16E+00	3.81E+00	0.410
Locus13660v1rpkm16.85	1.06E+01	5.54E+00	1.21E+01	6.09E+00	0.00E+00	9.88E+00	0.236
Locus1612v1rpkm125.79	9.22E+01	5.81E+00	1.50E+02	1.60E+02	2.79E+01	9.54E+01	0.225
Locus18090v1rpkm12.32	5.07E+00	1.87E+01	1.22E+01	1.39E+01	2.52E+01	6.41E+00	−0.465
Locus23870v1rpkm8.93	1.09E+01	0.00E+00	9.13E+00	2.82E+01	3.61E+00	1.86E+01	0.569
Locus2753v1rpkm80.23	7.20E+01	9.26E+00	1.29E+02	2.69E+02	3.68E+01	1.23E+02	0.352
Locus60938v1rpkm2.86	2.58E+00	4.46E-01	8.56E+00	6.57E+00	6.32E-01	5.24E+00	0.301
Locus611v1rpkm271.69	4.19E+02	4.06E+01	6.62E+02	1.12E+03	1.39E+02	5.98E+02	0.366
Locus611v2rpkm105.37	2.12E+02	1.92E+01	3.59E+02	4.10E+02	7.64E+01	2.83E+02	0.356
Locus614v1rpkm269.67	3.73E+01	2.41E+00	6.04E+01	4.90E+02	7.65E+01	2.93E+02	0.603
