# Supplementary material for: Transcriptome analysis of thermogenic Arum concinnatum reveals the molecular components of floral scent production
Source: Sci Rep. 2015 Mar 4;5:8753. doi: 10.1038/srep08753 (PMC5390080; doi:10.1038/srep08753)
Supplement: Supplementary Information — Supplementary Figures S1 and S2 [file srep08753-s5.pdf]

Supplementary Information File

Title: Transcriptome analysis of thermogenic *Arum concinnatum* reveals the molecular components of floral scent production

Yoshihiko Onda[1,2], Keiichi Mochida[1,2,3], Takuhiro Yoshida[4], Tetsuya Sakurai[4], Roger Seymour[5], Yui Umekawa[6], Stergios Arg. Pirintsos[7], Kazuo Shinozaki[1,3], Kikukatsu Ito[8]

[1]Biomass Research Platform Team, Biomass Engineering Program Cooperation Division, RIKEN Center for Sustainable Resource Science, Kanagawa, Japan

[2]Kihara Institute for Biological Research, Yokohama City University, Kanagawa, Japan

[3]Gene Discovery Research Group, RIKEN Center for Sustainable Resource Science, Kanagawa, Japan

[4]Integrated Genome Informatics Research Unit, RIKEN Center for Sustainable Resource Science, Kanagawa, Japan

[5]School of Biological Sciences, University of Adelaide, Australia

[6] United Graduate School of Agricultural Science, Iwate University, Morioka, Japan

[7]Department of Biology, University of Crete, Heraklion, Greece

[8]Cryobiofrontier Research Center, Iwate University, Morioka, Japan

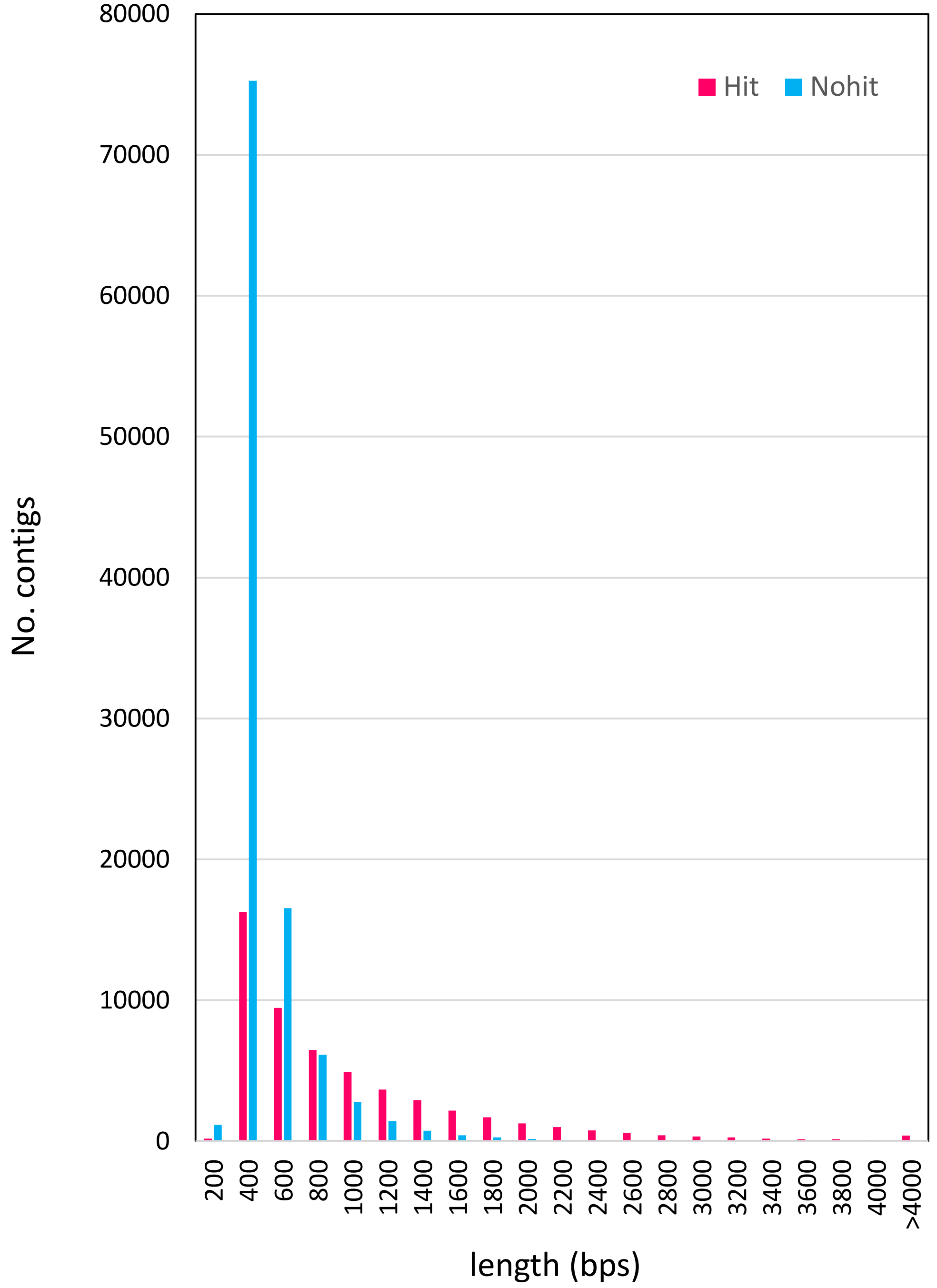

Supplementary Figure S1. Length distribution of assembled transcripts (contigs).

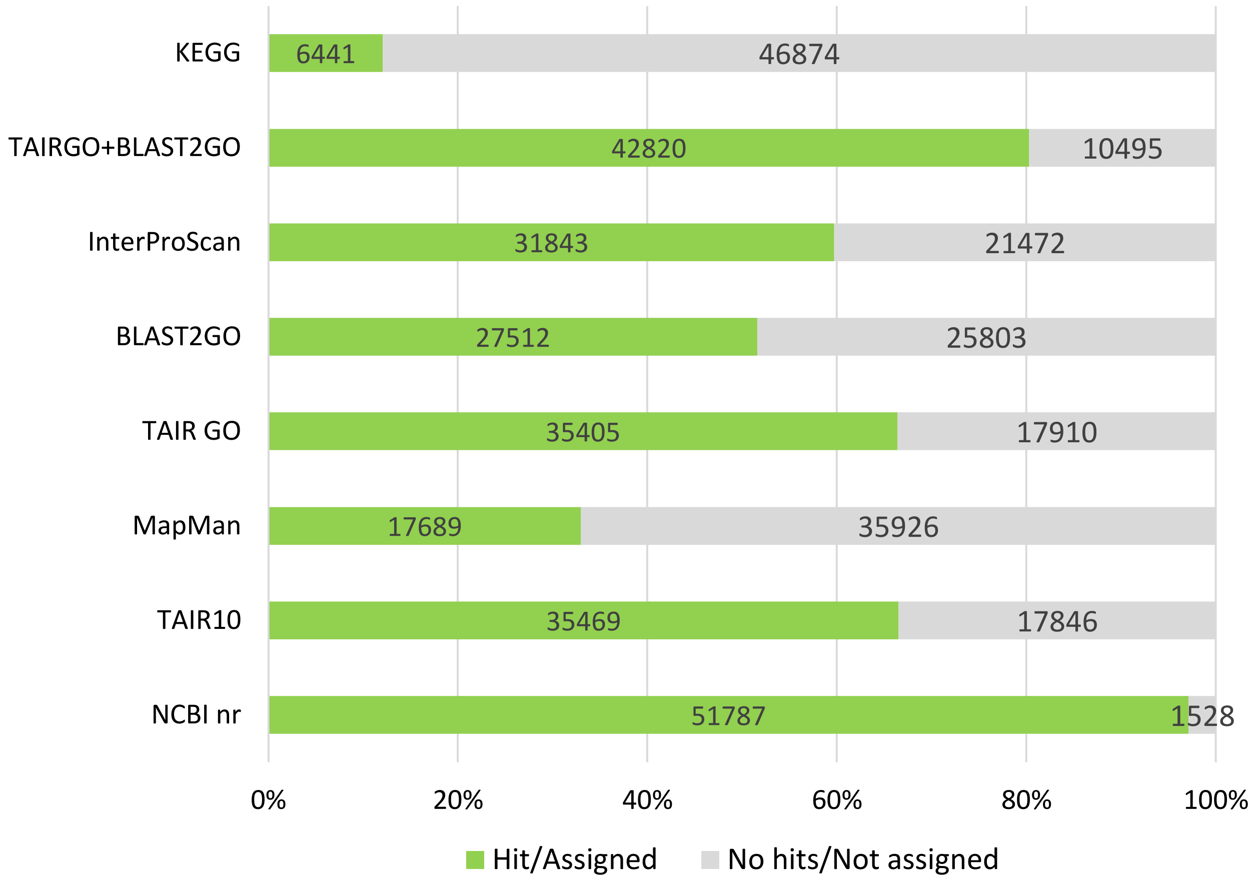

Supplementary Figure S2. Status of functional predication of gene models with various information resources.
